# The effect of camel urine on islet morphology and CCL4-induced liver cirrhosis in rat

**DOI:** 10.1186/1753-6561-6-S4-P42

**Published:** 2012-07-09

**Authors:** S Al Neyadi, R Al Jaberi, R Hameed, J Shafarin, E Adeghate

**Affiliations:** 1 Department of Anatomy, Faculty of Medicine & Health Sciences, United Arab Emirates

## Introduction

Camel urine has been used for decades as a medication for several ailments in the Middle East. Folklore medicine of the Middle East has shown that, camel urine has a beneficial effect in conditions such as liver cirrhosis.

## Methods

Camel urine was given as a drink daily to normal and treated rats for 4 weeks. Glucose tolerance test was performed at the end of the experiment. Immunohistochemistry was used to determine the percentage distribution of insulin and glucagon immunoreactive cells. H & E stain was used to access liver cirrhosis in control and urine-treated rats. 

## Results

The administration of camel urine significantly increased the number of insulin-positive cells in pancreatic islets. CCL4-treated rats did not have impaired glucose tolerance. CCL4 caused vacuolarization of hepatic cells. Rats treated with camel urine have improved hepatic morphology compared to untreated controls. 

## Conclusions

The study shows that camel urine may contain bioactive agents capable of preventing CCL4-induced hepatic and pancreatic islet lesions. 

